# The effect of the severity of obstructive sleep apnea syndrome on telomere length

**DOI:** 10.18632/oncotarget.12293

**Published:** 2016-09-27

**Authors:** Priscila Farias Tempaku, Diego Robles Mazzotti, Camila Hirotsu, Monica Levy Andersen, Gabriela Xavier, Pawan Kumar Maurya, Lucas Bortolotto Rizzo, Elisa Brietzke, Sintia Iole Belangero, Lia Bittencourt, Sergio Tufik

**Affiliations:** ^1^ Departamento de Psicobiologia, Universidade Federal de São Paulo (UNIFESP), São Paulo, Brasil; ^2^ Laboratório Interdisciplinar de Neurociências Clínicas (LINC), Universidade Federal de São Paulo (UNIFESP), São Paulo, Brasil; ^3^ Department of Psychiatry, University of Tuebingen, Tuebingen, Germany; ^4^ Grupo de Pesquisa em Neurociência Comportamental e Molecular do Transtorno Bipolar, Universidade Federal de São Paulo (UNIFESP), São Paulo, Brasil; ^5^ Departamento de Morfologia e Genética, Universidade Federal de São Paulo (UNIFESP), São Paulo, Brasil; ^6^ Amity Institute of Biotechnology, Amity University Uttar Pradesh, Noida, India

**Keywords:** sleep, telomeres, obstructive sleep apnea syndrome, aging, Gerotarget

## Abstract

Aging is associated with an increase in the prevalence of obstructive sleep apnea syndrome (OSAS) as well as the shortening of telomeres. It is known that OSAS-related factors are stimuli that can contribute to the acceleration of cellular senescence. Thus, the present study aimed to compare the leukocyte telomere length (LTL) between OSAS patients and controls, as well as to verify the correlation between LTL and sleep parameters. We used DNA extracted of 928 individuals from EPISONO to measure the LTL by the quantitative real-time polymerase chain reaction. All individuals were subjected to one full-night polysomnography. LTL was significantly shorter in OSAS patients compared to controls. The results showed negative correlations between LTL and the following variables: apnea-hypopnea index, respiratory disturbance index, desaturation index and wake after sleep onset. LTL was positively correlated with sleep efficiency, total sleep time, basal, minimum and maximum oxygen saturation. Lastly, it was observed that OSAS severity was associated with shorter LTL even after adjusting for sex, age, years of schooling, body mass index, diabetes, stroke and heart attack. In conclusion, our study indicates the presence of an association between LTL and OSAS and a significant impact of severity of OSAS in telomeres shortening.

## INTRODUCTION

Obstructive sleep apnea syndrome (OSAS) is a sleep related breathing disorder which is characterized by repetitive episodes of respiratory pauses (apnea) or partial upper airway obstruction (hypopnea). It is often associated with reduced blood oxygen saturation, snoring, sleep disruption and daytime sleepiness [1]. OSAS is known to stimulate oxidative stress, inflammation, sympathetic nervous system activation and endothelial dysfunction due mainly to sleep fragmentation and intermittent hypoxia mechanisms [2–7].

The core features of OSAS contribute to the exacerbation of inflammation and oxidative stress pathways [8]. Basic evidence has shown that intermittent hypoxia leads to recurrent blood oxygen desaturation, changes oxygen availability in many tissues, leading to an increase of pro-inflammatory cytokines and an imbalance between pro-oxidant and anti-oxidant systems resulting in excessive production of reactive oxygen species (ROS) [9]. Similarly, sleep fragmentation has been shown to promote increased expression and activation of nicotinamide adenine dinucleotide phosphate (NADPH) oxidase [5], an enzyme considered as major source of ROS generation in OSAS [8].

The accumulation of oxidative damage caused by ROS has an impact on critical aspects of aging process and contributes physiological function impairment, increased incidence of disease, and reduction in life span [10]. ROS molecules have the potential to damage a number of vital biomolecules [8]. At the cellular level, ROS leads to DNA double strand breaks and telomere shortening, which are both important cell senescence triggers [10].

Telomeres are characterized by the presence of tandem repeats of non-coding DNA (TTAGGG/CCCTAA) at the ends of chromosomes [11] and are responsible for protecting and stabilizing the terminal region of chromosomes, avoiding loss of coding DNA or chromosomal instability [12]. Telomere length tends to shorten with time, mainly due to the so called ‘end replication problem’, but also being due to other causes such as oxidative stress [12], genetic factors and comorbidities [13–15]. Telomeres are reported to be a ‘marker of biological aging’ since their length tends to diminish with age and be related to a higher risk of mortality [16]. Long telomeres are associated with decreased aging [13], while short telomeres are commonly found in several pathological conditions as an indicator of disease onset and related outcome [16].

Considering the fact that an important modulator of telomere maintenance is oxidative stress and that telomere-driven replicative senescence is primarily a stress response [17], evidences have pointed out to a potential association between OSAS and cellular aging, through telomere shortening [18–20]. Thus, we hypothesized that OSAS would be associated with shorter telomere length compared to control individuals without OSAS. Additionally, we aimed to assess the differences in the mean leukocyte telomere length (LTL) among mild, moderate and severe forms of OSAS as well as to verify the correlation between sleep parameters and LTL in an epidemiological framework.

## RESULTS

There were 613 individuals in the CTRL group and 315 in the OSAS group. Within the OSAS group, 48.6% were mild (5 ≤ AHI < 15), 31.1% moderate (15 ≤ AHI < 30) and 20.3% severe (AHI ≥ 30). Table 1 shows the clinical parameters investigated according to the presence or absence of OSAS in the EPISONO cohort. As expected, the OSAS group was older (*t* = −13.9, df = 926, *p* < 0.001), presented more men (χ^2^ = 25.872, df = 1, *p* < 0.001), had higher body mass index (*t* = −11.8, df = 926, *p* < 0.0019), respiratory disturbances index (RDI) (*t* = −21.4, df = 918, *p* < 0.001), AHI (*t* = −27.4, df = 926, *p* < 0.001), desaturation index (*t* = −18.5, df = 769, *p* < 0.001), REM sleep latency (*t* = −3.318, df = 917, *p* = 0.001), percentage of N1 (*t* = −4.420, df = 926, *p* < 0.001), N2 (*t* = −3.084, df = 926, *p* = 0.002), N3 (*t* = 3.889, df = 926, *p* < 0.001), wake after sleep onset (*t* = −5.450, df = 926, *p* < 0.001) and lower total sleep time (*t* = 3.245, df = 926, *p* < 0.001), sleep efficiency (*t* = 5.023, df = 926, *p* < 0.001), basal, medium and minimum SpO_2_ (*t* = 12.8, df = 925, *p* < 0.001; *t* = 13.9, df = 926, *p* < 0.001; *t* = 21.5, df = 926, *p* < 0.001, respectively) compared to the CTRL group.

**Table 1:** Non-adjusted mean ± standard deviation and frequency (%) of clinical characteristics from the EPISONO cohort according to the presence of obstructive sleep apnea syndrome

	CTRL	OSAS	*p*
	(*n* = 613)	(*n* = 315)	
**Age (years)**	38.4 ± 13	51.2 ± 13.7*	<0.001
**Men**	38.7%	56.2%*	<0.001
**BMI (kg/m**^2^**)**	25.5 ± 4.5	29.7 ± 5.9*	<0.001
**Years of Schooling**	10.84 ± 4.5	10.4 ± 5.3	0.195
**RDI**	2.3 ± 2.9	13.6 ± 12.5*	<0.001
**AHI**	1.9 ± 2.3	20.5 ± 16.5*	<0.001
**Basal SpO**_**2**_	96.3 ± 1.4	95 ± 1.5*	<0.001
**Medium SpO**_**2**_	95.7 ± 1.6	93.9 ± 1.9*	<0.001
**Minimum SpO**_**2**_	90.6 ± 3.7	83.7 ± 6.1*	<0.001
**Arousal Index**	11.36 ± 6.8	22.27 ± 14.2*	<0.001
**Sleep Latency (min)**	16.03 ± 21.2	17.65 ± 23.5	0.289
**REM Latency (min)**	97.22 ± 47.4	109.39 ± 61.6*	<0.001
**N1 (%)**	4.19 ± 3.2	5.18 ± 3.3*	<0.001
**N2 (%)**	54.00 ± 8.8	55.98 ± 10.1*	0.002
**N3 (%)**	22.5 ± 7.7	20.35 ± 8.6*	<0.001
**REM (%)**	19.29 ± 6.5	18.49 ± 6.6	0.078
**WASO**	53.43 ± 44.1	70.42 ± 46.5	<0.001
**TST**	348.72 ± 77.1	331.66 ± 73.3*	<0.001
**Sleep Efficiency**	83.48 ± 12.3	79.10 ± 13.1*	<0.001
**Dessaturation index**	1.9 ± 2.9	14.6 ± 14.3*	<0.001
**Presence of diabetes**	2.1%	9.5%*	<0.001
**History of heart attack**	1.3%	2.9%	0.201
**History of stroke**	1.1%	1.9%	0.554

CTRL: control; OSAS: obstructive sleep apnea syndrome; BMI: Body Mass Index; RDI: Respiratory Disturbances Index; AHI: Apnea-Hipopnea Index; REM: rapid eye movement; SpO_2_: Oxygen Saturation; TST: Total Sleep Time; WASO: Wake After Sleep Onset; p: Statistical Significance;

* statistically different compared to CTRL group.

Moreover, OSAS was associated with higher frequency of diabetes (χ^2^ = 25.977, df = 3, *p* < 0.001). However, no significant association between the comorbidities history of heart attack, and stroke was observed with OSAS (χ^2^ = 3.130, df = 2, *p* = 0.201; χ^2^ = 1.181, df = 2, *p* = 0.554; respectively), as well as no significant difference in years of schooling (*t* = 1.297, df = 922, *p* = 0.195), sleep latency (*t* = −1.062, df = 926, *p* = 0.289) and percentage of REM sleep (*t* = 1.767, df = 926, *p* = 0.07) between the groups being found.

**Figure 1:**
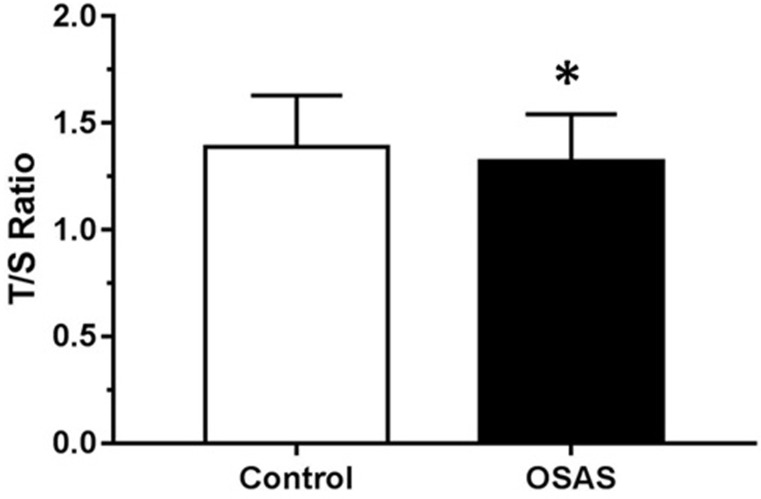
Mean telomere length expressed by T/S ratio among controls (CTRL) and obstructive sleep apnea syndrome (OSAS) group adjusted for age, body mass index and sex *p < 0.001 compared to CTRL group.

**Figure 2:**
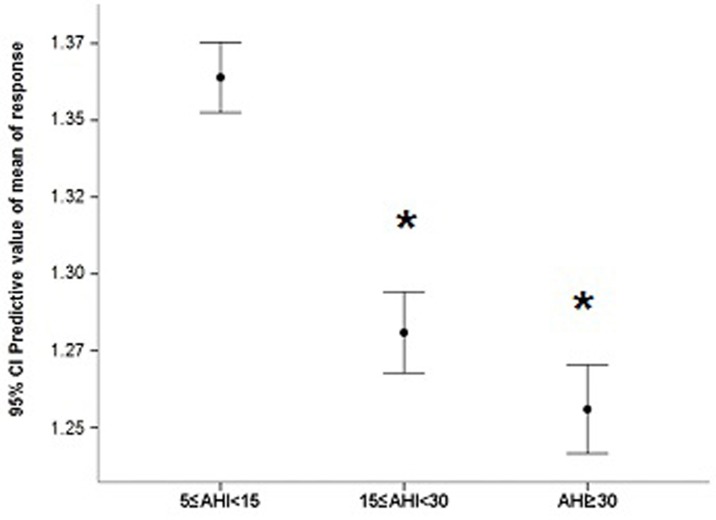
Mean predicted values for leukocyte telomere length adjusted for the covariates in the model (sex, age, years of schooling, body mass index, diabetes, stroke and heart attack) in different levels of obstructive sleep apnea syndrome severity according to apnea-hypopnea index (AHI) ranges *p < 0.05 compared to 5 < AIH < 15 (mild obstructive sleep apnea syndrome)

Figure 1 illustrates the comparison of LTL adjusted for confounders in individuals with OSAS and CTRL. As hypothesized, OSAS presented shorter LTL (mean = 1.32, SD = 0.22) than the CTRL (mean = 1.39, SD = 0.24) group (F = 27.010, df = 4, *p* < 0.001). The generalized linear model showed that in patients with OSAS (*n* = 315), OSAS severity (addressed by AHI) was associated with telomere length even after being adjusted for sex, age, body mass index, years of schooling, diabetes, stroke and heart attack (B = 0.055; CI = 0.007-0.102; *p* = 0.02). The post-hoc analysis showed that patients with mild OSAS had longer LTL (mean = 1.36, SD = 0.23) than those with severe OSAS (mean = 1.25, SD = 0.16). Moreover, patients with mild OSAS also had longer LTL than patients with moderate OSAS (mean = 1.28, SD = 0.20) (*p* = 0.02). No significant differences in LTL were observed between moderate and severe OSAS. Mean predicted values were calculated adjusted for the covariates in the model as shown in Figure 2. To address the relevance of the findings, Cohen's D effect size was calculated regarding the difference in telomere length according to age or to AHI. Between the age groups 20-29 years and 30-40 years, the effect size found in LTL was 0.17. In the comparison between CTRL and mild OSAS (5 ≤ AHI < 15), the effect size in LTL was −0.09; between CTRL and moderate OSAS (15 ≤ AHI < 30), it was 0.17; and between CTRL and severe OSAS (AHI ≥ 30), it was 0.21.

Table 2 demonstrates the correlation between LTL and polysomnographic parameters in the EPISONO cohort. The results showed negative correlations between LTL assessed by the average T/S ratio and the following variables: AHI, RDI, desaturation index, arousal index and wake after sleep onset. LTL was positively correlated with total sleep time, sleep efficiency, basal, minimum and medium SpO_2_. No correlations were observed between LTL and the following variables: sleep latency, REM sleep latency, percentages of N1, N2, N3 and REM sleep.

**Table 2:** Spearman's correlation between leukocyte telomere length and polysomnographic parameters in the EPISONO cohort

Polysomnographic Parameters	rho	*p*	*n*
**AHI**	−0.196	<0.001	928
**RDI**	−0.155	<0.001	920
**Dessaturation Index**	−0.182	<0.001	771
**Basal SpO**_**2**_	0.224	<0.001	927
**Medium SpO**_**2**_	0.235	<0.001	928
**Minimum SpO**_**2**_	0.200	<0.001	928
**Arousal Index**	−0.147	<0.001	928
**WASO**	−0.129	<0.001	928
**Sleep Efficiency**	0.127	<0.001	928
**TST**	0.074	0.023	928
**Sleep Latency**	−0.045	0.171	928
**REM Latency**	0.018	0.589	919
**N1**	0.060	0.069	928
**N2**	−0.035	0.287	928
**N3**	0.056	0.086	928
**REM**	0.019	0.567	928

AHI: Apnea-Hipopnea Index; RDI: Respiratory Disturbances Index; SpO_2_: Oxygen Saturation; TST: Total Sleep Time; WASO: Wake After Sleep Onset; rho: Spearman's Coefficient; p: Statistical Significance; n: Sample Size.

Table 3 represents the linear regression model considering as dependent variable T/S ratio. The results showed that age (β = 0.26, *t* = −7.46, *p* < 0.00) and medium SpO_2_ (β = 0.08, *t* = 2.32, *p* = 0.02) were predictors of telomere length in a model that explained about 98% of T/S variability. The variables excluded in the model were: gender, BMI, AHI, sleep efficiency and arousal index.

**Table 3:** Linear regression model considering T/S ratio as dependent variable and the sleep-related parameters as independent variables after controlling for age

	β	t	*p*
**Age**	0.26	−7.46	<0.001
**Medium SpO**_**2**_	0.08	2.32	0.02
**Gender**	0.06	1.75	0.08
**BMI**	0.04	1.03	0.30
**AHI**	−0.33	−0.91	0.36
**Sleep Efficiency**	−0.02	−0.044	0.66
**Arousal Index**	−0.02	−0.61	0.54

BMI: Body Mass Index; AHI: Apnea-Hipopnea Index; SpO_2_: Oxygen Saturation; β:regression coefficient; t: t statistic; p: Statistical Significance.

## DISCUSSION

The current study showed that OSAS was associated with decreased LTL in comparison to CTRL individuals in an epidemiological sample from Sao Paulo. We also found that LTL was correlated with sleep fragmentation and hypoxia-related parameters, being the last one, represented by mean SpO_2_, one of the most important predictors of LTL, after age. Although evidence from the literature had already pointed to a relationship between sleep disordered breathing and LTL [18–21], the present study has described the relationship between LTL and the main pathophysiological parameters of OSAS using objective methods in a representative population sample.

Barcelo and colleagues (2010) have also described shorter LTL in patients with OSAS compared to controls, even after adjustments for potential confounders. However, contrary to the findings of our study, the severity of OSAS did not show a relationship with LTL [22]. Savolainen et al. (2014) in a cross-sectional study investigated whether a history of OSAS or primary snoring had an association with LTL in later adulthood. Records derived from Helsinki Birth Cohort Study were used and the OSAS diagnoses were based on the International Classification of Diseases (ICD). It was found that participants with a history of possible OSAS had shorter LTL than controls, whereas individuals with a history of primary snoring did not differ in LTL from controls, suggesting that shorter LTL could be a specific risk factor for OSAS [23]. However, Kim et al. (2010) in a study of the relationship between telomere length and OSAS in children observed, in contrast to what had been expected, that children with OSAS had an increase in LTL compared to controls [24]. In order to explain this finding, the authors hypothesized that OSAS could induce early mobilization of mesenchymal stem cells and that this could have a protective effect when stimulated by hypoxia or inflammatory mediators in children. Moreover, patients with OSAS exhibited lymphocytes in a highly activated state and the fact that these cells can express telomerase could explain the preservation of LTL in children with OSAS [24]. In addition, the ages of the participants and the subjective instruments used to characterize OSAS in the Kim et al study, may help to explain the inconsistency found.

Regarding the results from the multivariate analysis, we observed that hypoxia was the most important OSAS-related factor associated with telomere length in our representative sample as we could see that medium SpO_2_ remained significant after controlling for the confounders, differently from the variables associated with sleep fragmentation, such as arousal index and sleep efficiency. In accordance with our results, Boyer and colleagues (2016) observed in a sample of middle-aged males that intermittent hypoxia, *via* oxygen dessaturation index, was an important contributor to telomere shortening, even controlling for confounders [18]. Similarly, Choi and colleagues found that OSA had a significant association with short telomere length, increasing the risk of brain white matter changes in a community-based cohort of aged people [20].

In respect of sleep duration, the literature suggests that short sleep duration is associated with shorter LTL [25–30]. However, this interpretation needs to be approached with some caution since these studies evaluated LTL and sleep in different sexes, age groups and health conditions. Furthermore, these studies relied on self-report questionnaires to asses sleep duration and sleep quality, which may have affected the quality of the data. Using data derived from polysomnography, we found a negative correlation of LTL with arousal index and wake after sleep onset. In contrast, there was a positive correlation between LTL and sleep efficiency, as well with total sleep time. These results confirm the relationship between short sleepers and reduced LTL and indicate that sleep duration may also play a role in LTL shortening.

The interpretation of the factors related to LTL should be done with caution since the relationship found is not always simple. It is known that long telomeres are associated with decreased aging of many forms, but long telomeres are also associated with several cancers [13]. It has already been established that LTL decreases with aging and that age-related diseases may play a role in the acceleration of this process. Moreover, cumulative levels of oxidative stress and inflammation may also influence telomere length [31]. In the present study, we found that LTL was shorter in OSAS individuals than in CTRL. We also observed correlations between sleep parameters which may suggest mechanisms for the relationship found between OSAS and LTL. Unlike the other studies, the severity of OSAS did show a relationship with LTL, even after adjusting for potential confounders.

Telomere length is a biomarker of aging very sensible to external stressors. Although a number of damage-causing mechanisms are needed to cause substantial changes in telomere length, as humans age, average telomere length declines and mortality increases. Thus, a small change in human white blood cell telomeres has a great functional impact than its absolute magnitude might suggest. The average change observed in LTL between controls and severe OSAS had an effect size of 0.21, an effect higher than the effect size regarding LTL in 2 groups with 10 years of age difference (0.17). Thus, we believe that OSAS may contribute to the attrition of telomere length and promote accelerated aging.

Despite the fact that the current study is cross-sectional, and thus causality cannot be inferred, the data found may encourage future prospective and experimental studies aiming to provide the possible molecular pathways underlying the relationship observed between OSAS and LTL, independent of aging. However, as a speculative hypothesis, we can argue that a possible mechanism relies on the cascade of events involving intermittent hypoxia/sleep fragmentation leading to increased production of ROS and pro-inflammatory cytokines, which are well-known factors able to shorten telomeres [22].

This study has some limitations. First, the cross-sectional design does not allow causality, only association. Second, there was no adaptation night for polysomnography. However, studies show good agreement between the first and second night of polysomnography for respiratory events, which were the main outcome in the current study [32]. Third, it is known that telomere length and its response to stresses varies by tissue, i.e., short-lived cells may be more expressive for genetic factors, whereas long-lived cells in other tissues may be more expressive for the effects of comorbidities such as OSAS [13].Thus, as in the current study the telomere length was measured in leukocytes (short-lived cells) for ethical reasons, we may assume that the results could be more expressive if done in other target cells.

Taken together, our findings showed that OSAS is associated with telomere shortening in AHI-dependent manner, increasing with disease severity. Moreover, in respect to OSAS-related factors, although sleep quality and duration are important, hypoxia seems to be a major contributor in the acceleration of telomere shortening. Therefore, the present study supports the idea of a possible relationship between the pathophysiology of OSAS and the molecular pathways of cell aging being related to the maintenance of telomere length. Prospective and experimental studies are essential to establish whether there is a causal relationship between OSAS and telomere shortening, and the possible mechanisms involved.

## MATERIALS AND METHODS

### Studied sample

This is part of a cross-sectional study called Sao Paulo Epidemiologic Sleep Study (EPISONO), a population-based survey, in which 1042 subjects underwent polysomnography and were investigated through sleep questionnaires and blood sample measurements. In order to adequately represent the inhabitants of Sao Paulo in 2007 according to gender, age (20-80 years), and socioeconomic class, a probabilistic three-stage cluster sample was used. A sample size was defined to allow for prevalence estimates with 3% precision [33]. The study protocol was approved by the Ethics Committee for Research of the Universidade Federal de São Paulo (CEP 0593/06) and registered with ClinicalTrials.gov (number: NCT00596713; name: Epidemiology of sleep disturbances among adult population of the Sao Paulo City). Written informed consent forms were completed and signed by all participants before their inclusion in the study.

### Polysomnography assessment

All individuals were subjected to 1 full-night polysomnography at the sleep laboratory using the EMBLA^®^ N7000 system (Embla Systems Inc., USA). Sleep stages were visually scored by trained technicians according to standardized criteria for investigating sleep [34, 35]. The exam was scheduled according to the volunteers' availability to try to respect their habitual sleep schedule, which was assessed through interview during home visit. Physiological variables evaluated during polysomnography included: electroencephalogram (4 channels: C3-A2, C4-A1, O1-A2, O2-A1); electrooculogram (2 channels: EOG-Left-A2, EOG-Right-A1); surface electromyogram (4 channels: submentonian region, masseter region, anterior tibialis muscle and seventh intercostal space); electrocardiogram (1 channel: derivation V1 modified); air flow (2 channels: thermocouple and nasal pressure); respiratory effort (2 channels: thorax and abdomen) by inductance plethysmography belts; snoring and body position (1 channel each) by EMBLA^®^ sensors; and oxygen saturation (SpO2) by EMBLA^®^ oximeter, which allowed the measurement of mean and minimum SpO_2_ both basal, when the patient was awake, and twice with the patient sleeping. The exam was performed according to specific criteria for the definition of sleep stages [34]. The American Academy of Sleep Medicine Manual for Scoring Sleep and Associated Events [35] was the reference used to score sleep-related respiratory events and arousals. Hypopneas were scored in accordance with the following rule: a decrease of 50% in the respiratory flow associated with arousal or 3% oxygen dessaturation [35]. The desaturation index comprised the number of incidents of 3% desaturation per hour of sleep, while the respiratory disturbance index was defined as the total number of obstructive respiratory events (apnea, hypopnea and respiratory effort related arousals).

### Diagnosis of obstructive sleep apnea syndrome

The present study included individuals with OSAS and controls. In accordance with the International Classification of Sleep Disorders (2005), OSAS was considered positive when individuals had an AHI of between 5 and 14.9, and had at least one of the following symptoms: loud snoring, daytime sleepiness, fatigue and respiratory cessations during sleep. Additionally, individuals with an AHI of 15 or above were also found positive, regardless of the presence of the above mentioned symptoms [36]. OSAS severity was addressed by apnea-hypopnea index, which classifies individuals with OSAS as: mild (5 ≤ AHI < 15), moderate (15 ≤ AHI < 30) and severe (AHI ≥ 30) [37]. Individuals that did not fulfill OSAS criteria were considered as controls (CTRL).

### Measurement of leukocyte telomere length

On the morning after the polysomnography, all volunteers had 10 mL of blood collected in EDTA tubes for DNA extraction. The DNA was extracted using the salting-out method, according to the Miller et al protocol [38]. After isolation, the DNA samples were quantified and diluted to 50 ng/ul. Telomere length measurement was performed by multiplex real time PCR, as described by Cawthon with some modifications [39]. Measurement consists of determining the relative ratio (T/S ratio) of ng of telomeres (T) to ng of albumin (single-copy gene, S) in experimental samples using a standard curve. The T/S ratio is proportional to the average telomere length.

A total of 928 samples were analyzed. There was a loss of a total of 114 samples due to: insufficient concentration (17 samples), insufficient volume (37 samples) and to the failure of some participants to fully complete the information questionnaire (60 samples). The final volume in the reaction wells was 25μL and contained: 12.5μL of 2x SYBR^®^ Select Master Mix (Life-Thermo Fischer Scientific, 4472920); 1μL sample DNA; 0.9μL of 25μM telg (final concentration 900nM); 0.6μL of 25μM telc (600nM); 0.9μL of 25μM albu (900nM); 0.9μL of 25μM albd (900nM) (20) and 8.2μL of UltraPure™ Dnase/RNase-Free Distilled Water (Life-Thermo Fischer Scientific, 10977035). The standard curve was built based on a five-point 1:3 serial dilution DNA from a single individual (range from 150 to 1.85ng). The samples and standard curve were all run in triplicate in a ViiA™ 7 Real-Time PCR System with fast 96-Well Block (Life-Thermo Fischer Scientific, USA).

The conditions of PCR amplifications were: 15 minutes at 95°C, 2 cycles of 15 seconds at 94°C, 15 seconds at 49°C, 35 cycles of 15 seconds at 94°C, 10 seconds at 68°C and 15 seconds at 74°C with acquisition of fluorescence (detection of telomere amplification), 10 seconds at 85°C, 15 seconds at 88°C with acquisition of fluorescence (detection of albumin amplification) and the melting curve.

In all runs, a seriated dilution from a standard DNA was added in order to construct a standard curve used to relatively quantify telomere length in relation to albumin gene (T/S Ratio) [39].

### Statistical analysis

Normality was determined by Kolmogorov-Smirnov's test. All variables that presented non-normal distribution were standardized by z-score before we ran an independent *T*-test on the simple comparisons between the OSAS and CTRL group. Chi-square test was used to verify possible associations between categorical variables, and consequently to identify confounding factors. Correlations between LTL (T/S Ratio) and polysomnographic parameters were examined using Spearman's correlation. In addition, the LTL comparison between the OSAS and CTRL groups was performed using analysis of covariance (ANCOVA), adjusted for the confounders age, body mass index and sex. Lastly, in order to determine the effect of OSAS severity (by AHI) on LTL, a generalized linear model (GLzMM) was used with Gamma distribution for sex, age, body mass index, years of schooling, diabetes, stroke and heart attack as independent variables (confounders) and T/S ratio as the dependent variable. Linear regression model was constructed for T/S ratio using the stepwise procedure. We considered as independent variables: age, gender, BMI, AHI, sleep efficiency, arousal index and medium SpO_2_. Furthermore, effect size for telomere length variance was calculated through the Cohen's D formula. Statistical significance was defined as 5%.
